# Metabolic Effects of Doxorubicin on the Rat Liver Assessed With Hyperpolarized MRI and Metabolomics

**DOI:** 10.3389/fphys.2021.782745

**Published:** 2022-01-05

**Authors:** Kerstin N. Timm, Vicky Ball, Jack J. Miller, Dragana Savic, James A. West, Julian L. Griffin, Damian J. Tyler

**Affiliations:** ^1^Department of Physiology, Anatomy and Genetics, University of Oxford, Oxford, United Kingdom; ^2^Department of Pharmacology, University of Oxford, Oxford, United Kingdom; ^3^Department of Physics, University of Oxford, Oxford, United Kingdom; ^4^Oxford Centre for Clinical Magnetic Resonance Research, John Radcliffe Hospital, Oxford, United Kingdom; ^5^The MR Research Center, The PET Centre, Aarhus University Hospital, Aarhus University, Aarhus, Denmark; ^6^Department of Biochemistry, University of Cambridge, Cambridge, United Kingdom; ^7^Department of Metabolism, Digestion and Reproduction, Faculty of Medicine, Imperial College London, London, United Kingdom

**Keywords:** hyperpolarized ^13^C, metabolomics, liver, doxorubicin, chemotherapy, toxicity, MRI

## Abstract

Doxorubicin (DOX) is a successful chemotherapeutic widely used for the treatment of a range of cancers. However, DOX can have serious side-effects, with cardiotoxicity and hepatotoxicity being the most common events. Oxidative stress and changes in metabolism and bioenergetics are thought to be at the core of these toxicities. We have previously shown in a clinically-relevant rat model that a low DOX dose of 2 mg kg^–1^ week^–1^ for 6 weeks does not lead to cardiac functional decline or changes in cardiac carbohydrate metabolism, assessed with hyperpolarized [1-^13^C]pyruvate magnetic resonance spectroscopy (MRS). We now set out to assess whether there are any signs of liver damage or altered liver metabolism using this subclinical model. We found no increase in plasma alanine aminotransferase (ALT) activity, a measure of liver damage, following DOX treatment in rats at any time point. We also saw no changes in liver carbohydrate metabolism, using hyperpolarized [1-^13^C]pyruvate MRS. However, using metabolomic analysis of liver metabolite extracts at the final time point, we found an increase in most acyl-carnitine species as well as increases in high energy phosphates, citrate and markers of oxidative stress. This may indicate early signs of steatohepatitis, with increased and decompensated fatty acid uptake and oxidation, leading to oxidative stress.

## Introduction

Doxorubicin (DOX) is a widely used chemotherapeutic agent for both solid and haematological malignancies. DOX’s cardiotoxic side-effects are well known (Moslehi, 2016) and limit the use of DOX despite its excellent anti-cancer action. The mechanism(s) for this toxicity are still poorly understood, although mitochondrial dysfunction has been implicated as a key contributor to cardiac functional decline (Tokarska-Schlattner et al., 2006). Hyperpolarized magnetic resonance imaging (MRI) and MR spectroscopy (MRS) can assess cardiac metabolic fluxes *in vivo* both in pre-clinical models and in patients (Timm et al., 2018). We have previously shown in a rat model of DOX-cardiotoxicity, that high dose DOX (3 mg kg^–1^ for 5 weeks) leads to cardiac systolic dysfunction, which is preceded by a decrease in cardiac carbohydrate metabolism, assessed by hyperpolarized [1-^13^C]pyruvate MRS. However, using a low dose of DOX (2 mg kg^–1^ for 6 weeks) we showed that there are no changes in cardiac carbohydrate metabolism and only mild cardiac systolic impairment, which were compensated by increased heart rate and thus led to normal cardiac index, a body-weighed adjusted measure of cardiac output (Timm et al., 2020).

However, cardiotoxicity is not the only side-effect of DOX, and hepatotoxicity associated with DOX-treatment in cancer-patients has also been known for decades (Aviles et al., 1984). DOX is metabolised in the liver by cytochrome P450 and carbonyl reductases (Kassner et al., 2008; Bains et al., 2010). The molecular mechanisms of DOX-induced hepatotoxicity are thought to be due to oxidative stress through reactive oxygen species (ROS) production, which occurs during DOX metabolism in the liver and which leads to inflammation and mitochondrial dysfunction (Prasanna et al., 2020). This eventually causes hepatocyte death and leakage of hepatic enzymes into the circulation. Therefore, DOX-induced hepatotoxicity has previously been assessed by liver serum biomarkers such as serum glutamic oxaloacetic transaminase (GOT) and serum glutamic pyruvic transaminase (GPT) in patients (Damodar et al., 2014) or alanine aminotransferase (ALT) in mice (Nagai et al., 2015). The aspartate aminotransferase (AST):ALT ratio is used in patients as part of the liver function test, and this should be <1 in healthy individuals. Nevertheless, these serum biomarkers indicate cellular damage, and thus a late stage in hepatotoxicity. Since mitochondrial dysfunction is thought to precede those changes, measurements of hepatocyte metabolism may offer an earlier indication of hepatotoxicity or give more assurance that a given dose of DOX is not causing mild liver toxicity.

Doxorubicin has been shown to increase adipose tissue lipolysis and decrease hepatic fatty acid metabolism, thereby inducing hepatosteatitis (Renu et al., 2019), which may lead to oxidative stress and cellular damage. Hyperpolarized magnetic resonance imaging (MRI) and spectroscopy (MRS) can be used to assess hepatic carbohydrate metabolism (Merritt et al., 2011), however, there are currently no markers of long chain fatty acid metabolism available for hyperpolarized ^13^C MRS. We therefore set out to assess if the serum biomarker ALT and hyperpolarized MRS of the *in vivo* liver can shed light on late and early hepatotoxic effects of DOX, respectively, in our low-dose rat model. We furthermore performed metabolomic analysis of liver tissue to assess both aqueous metabolites and acyl-carnitines, to complement carbohydrate metabolism MRS data with measurements of fatty acyl-CoAs to assess also lipid metabolism. This allowed us to measure not only *in vivo* real-time carbohydrate metabolism in the rat liver, but also generate a comprehensive list of metabolite changes, including fatty acyl-carnitines, thereby assessing early changes in fatty acid metabolism.

## Methods

### Animal Work

All animal experiments conformed to Home Office Guidance on the Operation of the Animals (Scientific Procedures) Act, 1986 and were approved by local and national ethical review. Age and weight-matched male Wistar rats (∼8 weeks, ∼250 g starting weight) were divided into two groups and treated weekly for 6 weeks with iv injections of either sterile saline (*n* = 12) or 2 mg kg^–1^ doxorubicin (Apollo Scientific) dissolved in sterile saline (DOX, *n* = 12). The volumes of both saline and DOX were matched. Rats were weighed twice weekly during the study. At weeks 1, 3, and 6 and at least 48 h after the preceding injection rats were anaesthetized with 2% isoflurane in oxygen (2 L/min) and underwent echocardiography and saphenous blood sampling. At these timepoints and during the same anaesthesia rats also underwent hyperpolarized [1-^13^C]pyruvate MRS. At the end of the week 6 scans rats were terminally anaesthetised (increase of isoflurane to 5%) and livers rapidly excised and freeze-clamped with liquid nitrogen-cooled Wollenberger tongs.

### Echocardiography

Transthoracic echocardiography was performed using a Vivid I echocardiography system (GE Healthcare) with an 11.5 MHz phased array 10S-RS paediatric probe. Left ventricular (LV) diameters were obtained from a short-axis view in M-mode at the level of the papillary muscles in diastole (LVd) and systole (LVs). LV ejection fraction (EF) was calculated as (LVd-LVs)/LVd × 100 [%] and fractional shortening was calculated as (LVd-LVs) × 100 [%]. 2D-guided pulsed-wave Doppler recordings of LV inflow were then obtained from the apical 4-chamber view to measure the ratio of the early diastolic velocity of mitral inflow (E) to the early diastolic velocity of mitral annular motion (E′), a preload independent reflection of left ventricular filling pressure.

### Aminotransferase Assay

Presence of alanine aminotransferase (ALT) indicative of hepatic damage was assayed in rat plasma from weeks 1, 3, and 6 using an ABX Pentra 400 clinical chemistry analyzer (Horiba ABX Diagnostics).

### Hyperpolarized Magnetic Resonance Spectroscopy

Real-time metabolic flux measurements in the liver were assessed with hyperpolarized [1-^13^C]pyruvate MRS, performed on a 7 T preclinical MRI system (Varian) as previously described (Timm et al., 2020). The [1-^13^C]pyruvate was polarised on an alpha prototype hyperpolarizer (Oxford Instruments) at 1.4 K for 35 min, until the build-up reached the plateau phase. Polarisation levels were not measured prior to injection. After dissolution, 1 mL of 80 mM [1-^13^C]pyruvate was manually injected into the tail vain over 10 s, ensuring a constant rate of injection as far as permissible by manual injection. A slice-selective MR spectrum (10 mm slab) was acquired from the liver region (180 acquisitions, 1 s TR, 15° Gaussian excitation pulse, 8 kHz bandwidth) using a 72 mm dual-tuned (^1^H/^13^C) birdcage volume transmit coil and a two-channel ^13^C surface receive coil (Rapid Biomedical). Multi-coil data were reconstructed *via* the whitened singular value decomposition method (Rodgers and Robson, 2010). The first 30 s of spectra following the appearance of the pyruvate peak were summed and quantified using AMARES/jMRUI as previously described (Vanhamme et al., 1997).

### Metabolomics

Metabolites were extracted from 50 mg samples of the freeze-clamped liver with 2:1 chloroform:methanol using metal-bead containing tubes and a Precellys tissue homogenizer (Bertin Instruments, Montigny-le-Bretonneux, France). Water was added to the extracts (20% v/v) and samples centrifuged to separate aqueous and lipid fractions. Metabolomic analysis of aqueous metabolites and acyl-carnitines was performed with liquid chromatography tandem mass spectrometry (LC-MS/MS) as previously described (Wang et al., 2015).

### Statistics

Echocardiography, hyperpolarized MRS and ALT data were analysed using a 2-way ANOVA with Tukey’s HSD correction for multiple comparisons. Metabolomic data were analysed using Student’s *t*-test (for each individual metabolite). Statistical significance was considered at *p* < 0.05.

## Results

### Low-Dose Doxorubicin Does Not Cause Liver Damage or Affect Cardiac Function in Rats

**FIGURE 1:**
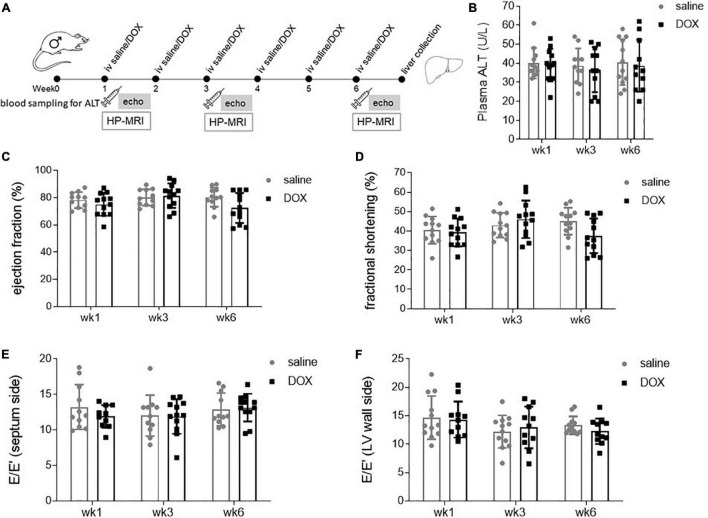
Low-dose doxorubicin does not cause liver damage or affect cardiac function in rats. **(A)** Study outline. Rats were treated weekly iv for 6 weeks with either sterile saline (*n* = 12) or 2 mg kg^– 1^ doxorubicin (*n* = 12). Two to four days after the first, third and last injection rats underwent echocardiography and saphenous blood sampling. At the 6 weeks timepoint rats also underwent hyperpolarized [1-^13^C]pyruvate MRS, after which rats were terminally anaesthetised and livers collected and freeze-clamped with liquid nitrogen-cooled Wollenberger tongs. **(B)** Plasma alanine aminotransferase (ALT) concentration. Echocardiography data displaying as systolic parameters: **(C)** cardiac left ventricular ejection fraction, **(D)** fractional shortening; and as diastolic parameters E/E′ of **(E)** septum and **(F)** wall side of the left ventricle. Data are shown as mean ± standard deviation.

Age and weight-matched male Wistar rats were treated for 6 consecutive weeks iv with either sterile saline (*n* = 12) or 2 mg kg^–1^ DOX (Figure 1A). At weeks 1, 3, and 6 there was no sign of liver damage, assessed by plasma ALT activity (Figure 1B). Rats also underwent echocardiography at weeks 1, 3, and 6, and this showed no significant changes in either systolic or diastolic function at any timepoint (Figures 1C–F).

### Low Dose Doxorubicin Does Not Affect Hepatic Carbohydrate Metabolism

**FIGURE 2:**
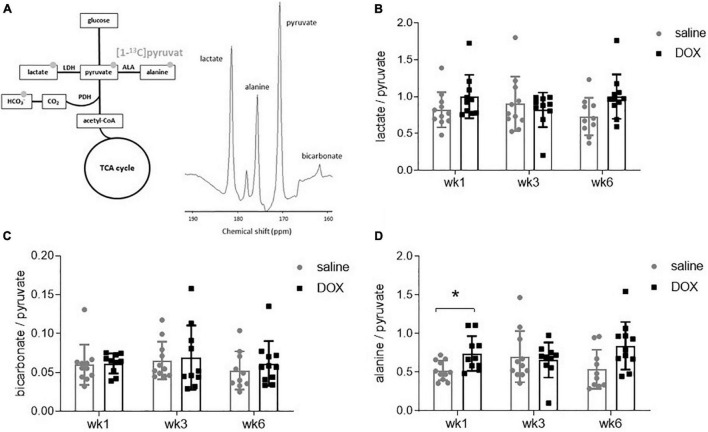
Hyperpolarized [1-^13^]pyruvate does not reveal alterations in hepatic carbohydrate metabolism after low-dose doxorubicin treatment. **(A)** All 24 rats received an injection of hyperpolarized [1-^13^]pyruvate at weeks 1, 3, and 6 for magnetic resonance spectroscopy of downstream metabolites. Example spectrum of 30 s of spectral data, starting from the first appearance of pyruvate, in a 10 mm slab covering the liver. The resonance between pyruvate and bicarbonate is a ^13^C urea phantom and the resonance between lactate and alanine is pyruvate hydrate, a break-down (non-metabolic) product of pyruvate. **(B)** Lactate:pyruvate ratio, **(C)** bicarbonate:pyruvate ratio and **(D)** alanine:pyruvate ratio. Data are shown as mean ± standard deviation. Statistical comparison was performed by 2-way ANOVA, with *post hoc* analysis using Tukey’s HSD for multiple comparisons. *p < 0.05.

All rats underwent hyperpolarized [1-^13^C]pyruvate MRS at weeks 1, 3, and 6. A 10 mm slab over the liver revealed resonances of [1-^13^C]pyruvate, as well as downstream metabolites [1-^13^C]lactate, [1-^13^C]bicarbonate and [1-^13^C]alanine (Figure 2A). The [1-^13^C]lactate signal was derived from ^13^C label-exchange between the endogenous lactate pool and injected hyperpolarized [1-^13^C]pyruvate through lactate dehydrogenase (LDH; Kettunen et al., 2010), [1-^13^C]bicarbonate was derived from oxidative decarboxylation of [1-^13^C]pyruvate through pyruvate dehydrogenase (PDH) (Schroeder et al., 2008) and [1-^13^C]alanine was derived through ALT activity from [1-^13^C]pyruvate. There were no changes in the metabolite to pyruvate ratio of either [1-^13^C]lactate or [1-^13^C]bicarbonate at any time point (Figures 2B–C). The average values and standard deviations for metabolite ratios at each timepoint were as follows: The lactate:pyruvate ratios for saline control and DOX were 0.82 ± 0.24 and 1.00 ± 0.29 at week 1; 0.91 ± 0.37 and 0.82 ± 0.24 at week 3, 0.73 ± 0.25 and 1.00 ± 0.30 at week 6, respectively. The bicarbonate:pyruvate ratios for saline control and DOX were 0.06 ± 0.3 and 0.06 ± 0.01 at week 1, 0.07 ± 0.02 and 0.07 ± 0.04 at week 3, 0.05 ± 0.3 and 0.06 ± 0.03 at week 6, respectively. At the week 1 timepoint there was a significant increase in [1-^13^C]alanine labelling in the DOX group (saline control 0.52 ± 0.13, DOX 0.74 ± 0.22), however, this was not seen at the other timepoints (week 3: saline control 0.70 ± 0.33, DOX 0.66 ± 0.23; week 6 saline control 0.54 ± 0.25, DOX 0.84 ± 0.31) (Figure 2D).

### Low Dose Doxorubicin Leads to Changes in Hepatic Metabolites and Increased Acyl-Carnitines

**FIGURE 3:**
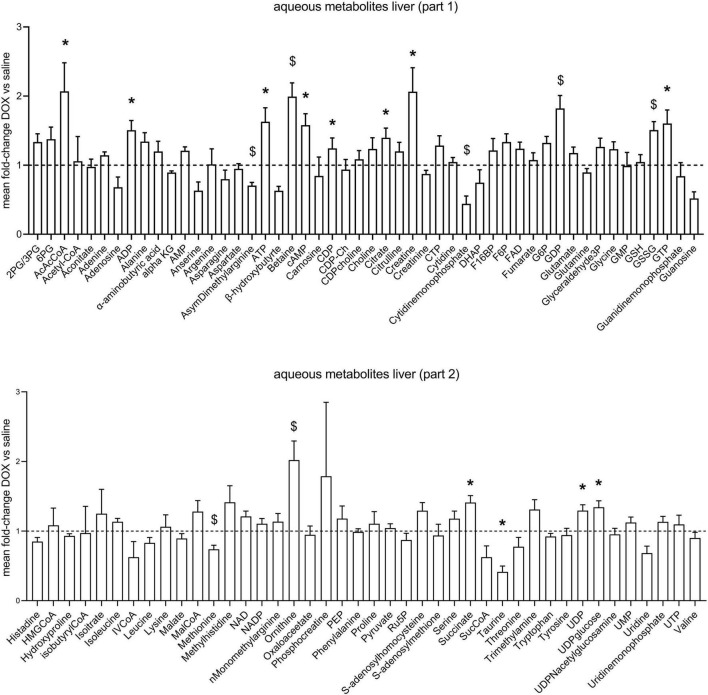
Low dose doxorubicin leads to changes in some hepatic metabolites. Aqueous metabolites in rat liver extracts. Data are shown as fold-change concentration compared to saline group (average concentration set to 1). Error bars indicate standard error of the mean. Group averages and standard deviations are given in Supplementary Table 1. Statistical comparison of individual metabolites was performed using student’s *t*-test. *p < 0.05, ^$^*p* < 0.01.

**FIGURE 4:**
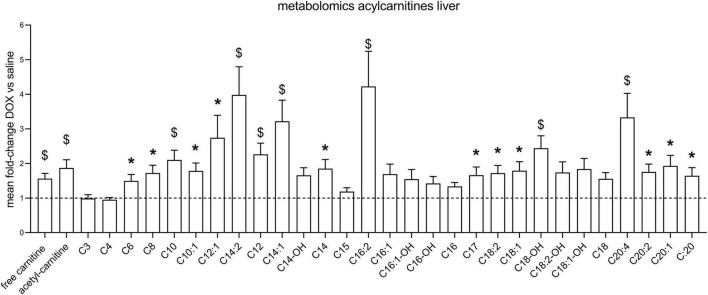
Low dose doxorubicin increases hepatic acyl-carnitines. Acyl-carnitine species in rat liver extracts. Data are shown as fold-change concentration compared to saline group (average concentration set to 1). Error bars indicate standard error of the mean. Group averages and standard deviations are given in Supplementary Table 2. Statistical comparison of individual metabolites was performed using student’s *t*-test. *p < 0.05, ^$^*p* < 0.01.

After the hyperpolarized MRS scans at week 6, rats were sacrificed and livers excised for metabolomic analysis. Supplementary Table 1 shows average values and standard deviations of all quantified aqueous metabolites in both the saline and DOX groups, and *p*-values are given for group comparisons (Student’s *t*-test). A total of 15 metabolites were increased in the DOX-treated liver [6-phosphogluconate, ADP, ATP, betaine, cAMP, CDP, citrate, creatine, GDP, oxidised glutathione (GSSG), GTP, ornithine, serine, UDP, and UDP-glucose], while only three were decreased with DOX (asymmetric dimethylarginine, cytidine monophosphate and methionine) (Figure 3). Supplementary Table 2 shows average values and standard deviations of all quantified acyl-carnitine species in both the saline and DOX groups, and *p*-values are given for group comparisons (Student’s *t*-test). There was an increase in free carnitine and acetyl-carnitine in the DOX group (Figure 4), as well as increases in most short chain (C3-C10), medium chain (C10-C15) and long chain (>C15) acyl-carnitines. None of the acyl-carnitine species were decreased upon DOX-treatment.

## Discussion

The main limitation of DOX is its severe cardiotoxicity. We have previously shown in a clinically-relevant rat model of DOX-cardiotoxicity, that hyperpolarized [1-^13^C]pyruvate MRS serves as an early detection tool for this toxicity (Timm et al., 2020), and that a low-dose of DOX does not lead to changes in cardiac carbohydrate metabolism. However, cardiotoxicity is not the only side-effect of DOX, with hepatotoxicity also being a major concern (Damodar et al., 2014). We therefore set out to assess liver metabolism in a low-dose DOX model to establish whether we would be able to observe any changes in hepatic metabolism at a dose that shows no cardiac dysfunction in rats. We found that at 2 mg kg^–1^ for 6 weeks, there was no significant decrease in cardiac function and that there was no indication of overt liver damage, assessed by plasma ALT activity. We could not detect an effect of low dose DOX on hepatic carbohydrate metabolism, using hyperpolarized [1-^13^C]pyruvate MRS, with the exception of an increased alanine:pyruvate ratio at week 1. However, power calculations determined that 15 rats would be required in each group to determine a difference in alanine:pyruvate ratio at 6 weeks with a power of 80% and α-error of 0.05. A larger cohort of animals may thus allow for small differences in hepatic alanine labelling from hyperpolarized [1-^13^C]pyruvate to be detected. Nevertheless, as MRS data show great variability, this unlikely to be a viable tool to assess early toxic effects of doxorubicin on the liver in patients.

Our metabolomic data revealed an increase in 6-phosphogluconate (6PG), an intermediate in the Pentose Phosphate Pathway (PPP), and an increase in oxidised glutathione (GSSG). Glutathione is a major intracellular antioxidant (Ballatori et al., 2009), and PPP-derived NADPH keeps the glutathione pool in its reduced (GSH) form (Couto et al., 2016). DOX-induced hepatotoxicity is thought to involve ROS production (Prasanna et al., 2020), and GSH can ‘scavenge’ those ROS by reducing them with glutathione peroxidase, while being itself oxidised to the GSSG disulfide (Couto et al., 2016). An increase in 6PG may thus represent an attempt to counteract oxidation of GSH to GSSG by ROS, and thus reflect early signs of toxic damage on the liver. In fact, supplementation of cystathionine as a glutathione precursor has previously been shown to alleviate DOX-induced hepatotoxicity (Kwiecień et al., 2006).

We furthermore found an increase in hepatic creatine and a decrease in methionine. Creatine is synthesised in the kidney and liver, but the last step, methylation of guanidinoacetate acid, which requires methionine, is mostly performed in the liver (Da Silva et al., 2009). An increase in creatine and a decrease in methionine may therefore reflect increased hepatic creatine synthesis. Livers from DOX-treated rats also had increased ornithine concentrations, which is part of the hepatic urea cycle (nitrogen excretion). Rats on DOX-treatment lose weight over time (Timm et al., 2020), and an increase in hepatic urea cycle may reflect enhanced protein breakdown and nitrogen excretion.

Surprisingly, we found an increase in high energy phosphates in livers from DOX-treated rats (ADP, ATP, GDP, GTP, UDP), as well as an increase in citrate. This may reflect an increase in TCA-cycle activity. Since we saw no changes in PDH flux in these rats using hyperpolarized MRS, suggesting no change in carbohydrate oxidation, which may reflect an increased proportion of fatty acid oxidation contributing to ATP synthesis. Interestingly, we also found that most acyl-carnitine species were increased in the liver. Fatty acids enter the mitochondria for oxidation in the form of acyl-carnitine esters (Longo et al., 2016), and an increase in these acyl-carnitines may explain higher liver energetics and citrate levels. However, elevation of plasma acyl-carnitines (especially long-chain) are also associated with non-alcoholic fatty liver disease (NAFLD) in patients (Enooku et al., 2019), and an increase of long-chain acyl-carnitines in liver tissue is associated with non-alcoholic steatohepatitis (NASH; Pérez-Carreras et al., 2003). An increase in acyl-carnitines in livers of DOX treated rats may therefore also reflect early signs of steatohepatitis. We furthermore found increased concentrations of aceto-acetyl-CoA (AcAcCoA), a metabolite in ketone body synthesis. Ketone bodies are made in the liver when there is excess oxidation of fatty acids, and an increase in AcAcCoA may thus support the notion that increased levels of acyl-carnitines are maladaptive in DOX-treated rats, signalling early signs of fatty liver disease.

Overall, we have shown that a low dose of DOX that shows no cardiac side effects in rats appears to also not cause overt liver cellular damage or changes in carbohydrate metabolism that could be observed with hyperpolarized [1-^13^C]pyruvate MRS. However, we found that low-dose of DOX leads to an increase in antioxidant-defence mechanisms in the liver, reflecting potential early oxidative damage. We also found an increase in most hepatic acyl-carnitine species with DOX-treatment, which may reflect increased adipose lipolysis and hepatic fatty acid uptake. Since we also saw a high energy state and increased citrate concentrations in those livers, this suggests that fatty acid oxidation may be enhanced in the liver upon DOX treatment. This may be compensatory, but since DOX treatment can lead to steatohepatitis, increased hepatic acyl-carnitine species may serve as an early sign of excess lipid accumulation in the liver.

## Data Availability Statement

The original contributions presented in the study are included in the article/Supplementary Material, further inquiries can be directed to the corresponding author.

## Ethics Statement

The animal study was reviewed and approved by the Animal Care and Ethical Review Committee, University of Oxford. All animal experiments conformed to Home Office Guidance on the Operation of the Animals (Scientific Procedures) Act, 1986.

## Author Contributions

KT conceptualized and designed the study, performed all experiments, analysed the data, and wrote the manuscript. VB helped with all MR imaging experiments. JM helped with some MRI experiments and edited the manuscript. DS helped with some MRI and echocardiography experiments and edited the manuscript. JW helped perform and analyse metabolomics data. JG helped design metabolomics experiments and data analysis. DT contributed to conception and design of the study and edited the manuscript. All authors contributed to the article and approved the submitted version.

## Conflict of Interest

The authors declare that the research was conducted in the absence of any commercial or financial relationships that could be construed as a potential conflict of interest.

## Publisher’s Note

All claims expressed in this article are solely those of the authors and do not necessarily represent those of their affiliated organizations, or those of the publisher, the editors and the reviewers. Any product that may be evaluated in this article, or claim that may be made by its manufacturer, is not guaranteed or endorsed by the publisher.

## References

[B1] Aviles A. Herrera J. Ramos E. Ambriz R. Aguirre J. Pizzuto J. (1984). Hepatic injury during doxorubicin therapy. *Arch. Pathol. Lab. Med.* 108 912–913. 6548368

[B2] Bains O. S. Grigliatti T. A. Reid R. E. Riggs K. W. (2010). Naturally occurring variants of human aldo-keto reductases with reduced in vitro metabolism of daunorubicin and doxorubicin. *J. Pharmacol. Exp. Ther.* 335 533–545. 10.1124/jpet.110.173179 20837989

[B3] Ballatori N. Krance S. M. Notenboom S. Shi S. Tieu K. Hammond C. L. (2009). Glutathione dysregulation and the etiology and progression of human diseases. *Biol. Chem.* 390 191–214. 10.1515/BC.2009.033 19166318 PMC2756154

[B4] Couto N. Wood J. Barber J. (2016). The role of glutathione reductase and related enzymes on cellular redox homoeostasis network. *Free Radic. Biol. Med.* 95 27–42. 10.1016/j.freeradbiomed.2016.02.028 26923386

[B5] Da Silva R. P. Nissim I. Brosnan M. E. Brosnan J. T. (2009). Creatine synthesis: hepatic metabolism of guanidinoacetate and creatine in the rat in vitro and in vivo. *Am. J. Physiol. Endocrinol. Metab.* 296 E256–E261. 10.1152/ajpendo.90547.2008 19017728 PMC2645018

[B6] Damodar G. Smitha T. Gopinath S. Vijayakumar S. Rao Y. (2014). An evaluation of hepatotoxicity in breast cancer patients receiving injection doxorubicin. *Ann. Med. Health Sci. Res.* 4 74–79. 10.4103/2141-9248.126619 24669335 PMC3952301

[B7] Enooku K. Nakagawa H. Fujiwara N. Kondo M. Minami T. Hoshida Y. (2019). Altered serum acylcarnitine profile is associated with the status of nonalcoholic fatty liver disease (NAFLD) and NAFLD-related hepatocellular carcinoma. *Sci. Rep.* 9:10663. 10.1038/s41598-019-47216-2 31337855 PMC6650415

[B8] Kassner N. Huse K. Martin H. J. Gödtel-Armbrust U. Metzger A. Meineke I. (2008). Carbonyl reductase 1 is a predominant doxorubicin reductase in the human liver. *Drug Metab. Dispos.* 36 2113–2120. 10.1124/dmd.108.022251 18635746

[B9] Kettunen M. I. Hu D. E. Witney T. H. McLaughlin R. Gallagher F. A. Bohndiek S. E. (2010). Magnetization transfer measurements of exchange between hyperpolarized [1-13C]pyruvate and [1-13C]lactate in a murine lymphoma. *Magn. Reson. Med.* 63 872–880. 10.1002/mrm.22276 20373388

[B10] Kwiecień I. Michalska M. Włodek L. (2006). The selective effect of cystathionine on doxorubicin hepatotoxicity in tumor-bearing mice. *Eur. J. Pharmacol.* 550 39–46. 10.1016/j.ejphar.2006.09.001 17034787

[B11] Longo N. Frigeni M. Pasquali M. (2016). Carnitine transport and fatty acid oxidation. *Biochim. Biophys. Acta* 1863 2422–2435. 10.1016/j.bbamcr.2016.01.023 26828774 PMC4967041

[B12] Merritt M. E. Harrison C. Sherry A. D. Malloy C. R. Burgess S. C. (2011). Flux through hepatic pyruvate carboxylase and phosphoenolpyruvate carboxykinase detected by hyperpolarized 13C magnetic resonance. *Proc. Natl. Acad. Sci. U. S. A.* 108 19084–19089. 10.1073/pnas.1111247108 22065779 PMC3223470

[B13] Moslehi J. J. (2016). Cardiovascular toxic effects of targeted cancer therapies. *N. Engl. J. Med.* 375 1457–1467. 10.1056/NEJMra1100265 27732808

[B14] Nagai K. Oda A. Konishi H. (2015). Theanine prevents doxorubicin-induced acute hepatotoxicity by reducing intrinsic apoptotic response. *Food Chem. Toxicol.* 78 147–152. 10.1016/j.fct.2015.02.009 25680506

[B15] Pérez-Carreras M. Del Hoyo P. Martín M. A. Rubio J. C. Martín A. Castellano G. (2003). Defective hepatic mitochondrial respiratory chain in patients with nonalcoholic steatohepatitis. *Hepatology* 38 999–1007. 10.1053/jhep.2003.50398 14512887

[B16] Prasanna P. L. Renu K. Valsala Gopalakrishnan A. (2020). New molecular and biochemical insights of doxorubicin-induced hepatotoxicity. *Life Sci.* 250:117599. 10.1016/j.lfs.2020.117599 32234491

[B17] Renu K. Sruthy K. B. Parthiban S. Sugunapriyadharshini S. George A. Tirupathi T. P. (2019). Elevated lipolysis in adipose tissue by doxorubicin via PPARα activation associated with hepatic steatosis and insulin resistance. *Eur. J. Pharmacol.* 843 162–176. 10.1016/j.ejphar.2018.11.018 30452912

[B18] Rodgers C. T. Robson M. D. (2010). Receive array magnetic resonance spectroscopy: whitened singular value decomposition (WSVD) gives optimal bayesian solution. *Magn. Reson. Med.* 63 881–891. 10.1002/mrm.22230 20373389

[B19] Schroeder M. A. Cochlin L. E. Heather L. C. Clarke K. Radda G. K. Tyler D. J. (2008). In vivo assessment of pyruvate dehydrogenase flux in the heart using hyperpolarized carbon-13 magnetic resonance. *Proc. Natl. Acad. Sci. U. S. A.* 105 12051–12056. 10.1073/pnas.0805953105 18689683 PMC2515222

[B20] Timm K. N. Miller J. J. Henry J. A. Tyler D. J. (2018). Cardiac applications of hyperpolarised magnetic resonance. *Prog. Nucl. Magn. Reson. Spectrosc.* 106–107 66–87. 10.1016/j.pnmrs.2018.05.002 31047602

[B21] Timm K. N. Perera C. Ball V. Henry J. A. Miller J. J. Kerr M. (2020). Early detection of doxorubicin-induced cardiotoxicity in rats by its cardiac metabolic signature assessed with hyperpolarized MRI. *Commun. Biol*. 3:692. 10.1038/s42003-020-01440-z 33214680 PMC7678845

[B22] Tokarska-Schlattner M. Zaugg M. Zuppinger C. Wallimann T. Schlattner U. (2006). New insights into doxorubicin-induced cardiotoxicity: the critical role of cellular energetics. *J. Mol. Cell. Cardiol.* 41 389–405. 10.1016/j.yjmcc.2006.06.009 16879835

[B23] Vanhamme L. Van Den Boogaart A. Van Huffel S. (1997). Improved method for accurate and efficient quantification of MRS data with use of prior knowledge. *J. Magn. Reson.* 129 35–43. 10.1006/jmre.1997.1244 9405214

[B24] Wang X. West J. A. Murray A. J. Griffin J. L. (2015). Comprehensive metabolic profiling of age-related mitochondrial dysfunction in the high-fat-fed ob/ob mouse heart. *J. Proteome Res.* 14 2849–2862. 10.1021/acs.jproteome.5b00128 25985803

