# Supplementary material for: Metabolic Effects of Doxorubicin on the Rat Liver Assessed With Hyperpolarized MRI and Metabolomics
Source: Front Physiol. 2022 Jan 5;12:782745. doi: 10.3389/fphys.2021.782745 (PMC8766499; doi:10.3389/fphys.2021.782745)
Supplement: Supplementary file 1 [file Table_1.DOCX]

**Supplementary Table 1: Metabolomics of aqueous metabolites in liver tissue extracts**

| **Metabolite** | **Mean conc. (mM) saline ctrl** | **Std. dev. (mM) saline ctrl** | **Mean conc. (mM) DOX** | **Std. dev. (mM) DOX** | **p-value DOX vs saline (Student’s t-test)** |
| --- | --- | --- | --- | --- | --- |
| 2PG/3PG | 0.259444 | 0.113544 | 0.346438 | 0.101893 | 0.071832 |
| 6PG | 0.008667 | 0.004379 | 0.011921 | 0.005025 | 0.119618 |
| AcAcCoA | 0.000121 | 0.000106 | 0.000249 | 0.000165 | 0.04043 |
| Acetyl-CoA | 0.001623 | 0.002063 | 0.001716 | 0.001921 | 0.91379 |
| Aconitate | 0.141308 | 0.066289 | 0.137978 | 0.051883 | 0.896803 |
| Adenine | 0.005648 | 0.001344 | 0.006453 | 0.000911 | 0.114239 |
| Adenosine | 0.039589 | 0.077123 | 0.026946 | 0.019468 | 0.603356 |
| ADP | 2.284727 | 0.880898 | 3.442549 | 1.055611 | 0.010602 |
| Alanine | 0.255718 | 0.1163 | 0.342829 | 0.108942 | 0.083495 |
| α-aminobutyric acid | 0.000222 | 7.11E-05 | 0.000265 | 0.000109 | 0.274734 |
| alpha KG | 0.142962 | 0.02475 | 0.127619 | 0.011939 | 0.077525 |
| AMP | 4.722561 | 2.361058 | 5.712125 | 0.879513 | 0.206177 |
| Anserine | 0.021707 | 0.042629 | 0.013692 | 0.009029 | 0.548038 |
| Argenine | 0.05287 | 0.020287 | 0.053567 | 0.039117 | 0.958641 |
| Asparagine | 0.001586 | 0.000524 | 0.001267 | 0.000685 | 0.232664 |
| Aspartate | 0.048744 | 0.010924 | 0.046185 | 0.012029 | 0.606561 |
| AsymDimethylarginine | 0.000135 | 3.06E-05 | 9.51E-05 | 2.05E-05 | 0.00162 |
| ATP | 4.150341 | 2.26837 | 6.761933 | 2.776428 | 0.024444 |
| β-hydroxybutyrte | 0.002688 | 0.00177 | 0.001692 | 0.000575 | 0.089741 |
| Betaine | 0.738854 | 0.268744 | 1.471604 | 0.4883 | 0.00025 |
| cAMP | 0.028054 | 0.012655 | 0.044291 | 0.015477 | 0.013268 |
| Carnosine | 0.007237 | 0.011856 | 0.006105 | 0.006551 | 0.784261 |
| CDP | 0.009473 | 0.00236 | 0.012844 | 0.00331 | 0.02028 |
| CDP-Ch | 0.13554 | 0.047329 | 0.126968 | 0.065128 | 0.727375 |
| CDPcholine | 0.004063 | 0.001115 | 0.004411 | 0.001686 | 0.573928 |
| Choline | 0.031698 | 0.01427 | 0.039142 | 0.017056 | 0.278855 |
| Citrate | 0.546785 | 0.235637 | 0.764017 | 0.253938 | 0.049407 |
| Citrulline | 0.109767 | 0.032881 | 0.131618 | 0.047499 | 0.222818 |
| Creatine | 0.005695 | 0.003764 | 0.011758 | 0.006528 | 0.014045 |
| Creatinine | 0.00772 | 0.002327 | 0.006741 | 0.001341 | 0.23934 |
| CTP | 0.008109 | 0.004706 | 0.010413 | 0.003784 | 0.21889 |
| Cytidine | 0.00334 | 0.001385 | 0.003494 | 0.000711 | 0.745907 |
| Cytidinemonophosphate | 0.003951 | 0.002049 | 0.001742 | 0.001468 | 0.008189 |
| DHAP | 0.001276 | 0.001615 | 0.001041 | 0.000763 | 0.677974 |
| F16BP | 0.074929 | 0.065431 | 0.090818 | 0.042993 | 0.507902 |
| F6P | 0.259444 | 0.113544 | 0.346438 | 0.101893 | 0.071832 |
| FAD | 0.640374 | 0.259696 | 0.792535 | 0.201762 | 0.139147 |
| Fumarate | 0.009319 | 0.003791 | 0.010016 | 0.00324 | 0.647669 |
| G6P | 0.518665 | 0.254005 | 0.685456 | 0.161633 | 0.079698 |
| GDP | 0.01307 | 0.007251 | 0.023806 | 0.008077 | 0.003418 |
| Glutamate | 2.588832 | 0.744497 | 3.044764 | 0.741506 | 0.164199 |
| Glutamine | 3.884394 | 0.93234 | 3.47655 | 0.709345 | 0.260627 |
| Glyceraldehyde3P | 0.004712 | 0.002445 | 0.005961 | 0.001952 | 0.199255 |
| Glycine | 0.001494 | 0.000267 | 0.001839 | 0.000527 | 0.065653 |
| GMP | 0.048768 | 0.058318 | 0.048188 | 0.031614 | 0.97712 |
| GSH | 0.001524 | 0.00057 | 0.001594 | 0.000541 | 0.77116 |
| GSSG | 0.426552 | 0.141654 | 0.642882 | 0.173603 | 0.004111 |
| GTP | 0.066986 | 0.039552 | 0.107433 | 0.043175 | 0.031905 |
| Guanidinemonophosphate | 0.01 | 0.012286 | 0.008403 | 0.006507 | 0.706856 |
| Guanosine | 0.000575 | 0.000857 | 0.000298 | 0.000182 | 0.305375 |
| Histadine | 0.719343 | 0.212233 | 0.610237 | 0.141111 | 0.169672 |
| HMGCoA | 0.004533 | 0.004484 | 0.004905 | 0.003732 | 0.834753 |
| Hydroxyproline | 0.028203 | 0.006701 | 0.026237 | 0.002996 | 0.384112 |
| isobutyrylCoA | 0.000359 | 0.0004 | 0.000348 | 0.000458 | 0.954564 |
| Isoitrate | 0.026211 | 0.025726 | 0.032719 | 0.030641 | 0.594964 |
| Isoleucine | 0.059404 | 0.014173 | 0.067356 | 0.009593 | 0.137611 |
| IVCoA | 0.000699 | 0.00086 | 0.000436 | 0.000522 | 0.395088 |
| Leucine | 0.130377 | 0.029631 | 0.10824 | 0.033662 | 0.115822 |
| Lysine | 0.17045 | 0.041161 | 0.217189 | 0.058107 | 0.0753 |
| Malate | 7.039173 | 3.088731 | 6.290354 | 1.631011 | 0.484539 |
| MalCoA | 0.000171 | 0.000101 | 0.000219 | 9.04E-05 | 0.252416 |
| Methionine | 0.003671 | 0.000745 | 0.002709 | 0.000718 | 0.005452 |
| Methylhistidine | 0.011127 | 0.002224 | 0.015725 | 0.008798 | 0.122375 |
| NAD | 1.962182 | 0.562831 | 2.373329 | 0.504642 | 0.084962 |
| NADP | 0.343414 | 0.147688 | 0.37957 | 0.085892 | 0.490108 |
| nMonomethylarginine | 0.007254 | 0.001915 | 0.008236 | 0.002825 | 0.350242 |
| Ornithine | 0.061078 | 0.014377 | 0.123329 | 0.055541 | 0.001596 |
| Oxaloaceetate | 0.012507 | 0.006582 | 0.011825 | 0.005292 | 0.791215 |
| Phosphocreatine | 0.146649 | 0.165599 | 0.262046 | 0.51725 | 0.488392 |
| PEP | 0.375121 | 0.176532 | 0.442423 | 0.225114 | 0.443551 |
| Phenylalanine | 0.049876 | 0.010342 | 0.049221 | 0.007621 | 0.867134 |
| Proline | 0.092138 | 0.020674 | 0.101774 | 0.053461 | 0.582787 |
| Pyruvate | 0.000129 | 4.43E-05 | 0.000135 | 2.66E-05 | 0.728987 |
| Ru5P | 0.007374 | 0.009305 | 0.006422 | 0.00239 | 0.745516 |
| S-adenosylhomocysteine | 0.000179 | 4.99E-05 | 0.000232 | 6.97E-05 | 0.055225 |
| S-adenosylmethione | 0.002373 | 0.001624 | 0.00222 | 0.001277 | 0.808627 |
| Serine | 0.022223 | 0.006908 | 0.026158 | 0.008166 | 0.235396 |
| Succinate | 0.078257 | 0.029771 | 0.110251 | 0.026121 | 0.013704 |
| SucCoA | 0.00054 | 0.000508 | 0.000336 | 0.000293 | 0.261859 |
| Taurine | 0.013755 | 0.011444 | 0.005701 | 0.003829 | 0.037531 |
| Threonine | 0.058832 | 0.019101 | 0.045592 | 0.026108 | 0.188409 |
| Trimethylamine | 0.001481 | 0.000633 | 0.00194 | 0.000693 | 0.119127 |
| Tryptophan | 0.009957 | 0.001695 | 0.009184 | 0.001439 | 0.26111 |
| Tyrosine | 0.021832 | 0.008714 | 0.02056 | 0.007096 | 0.710945 |
| UDP | 0.181605 | 0.047815 | 0.234906 | 0.051181 | 0.019329 |
| UDPglucose | 12.23835 | 3.823306 | 16.42348 | 3.745553 | 0.016587 |
| UDPNacetylglucosamine | 14.16229 | 5.747159 | 13.48188 | 4.145619 | 0.753147 |
| UMP | 0.032937 | 0.011822 | 0.036939 | 0.008716 | 0.375876 |
| Uridine | 0.000673 | 0.001272 | 0.000459 | 0.000226 | 0.587994 |
| Uridinemonophosphate | 0.01011 | 0.004286 | 0.011432 | 0.002685 | 0.395366 |
| UTP | 0.342962 | 0.202231 | 0.375302 | 0.152596 | 0.676139 |
| Valine | 0.050353 | 0.012376 | 0.045308 | 0.013725 | 0.375053 |
