# Supplementary material for: Metabolic Effects of Doxorubicin on the Rat Liver Assessed With Hyperpolarized MRI and Metabolomics
Source: Front Physiol. 2022 Jan 5;12:782745. doi: 10.3389/fphys.2021.782745 (PMC8766499; doi:10.3389/fphys.2021.782745)
Supplement: Supplementary file 2 [file Table_2.DOCX]

**Supplementary Table 2: Metabolomics of acyl-carnitine species in liver tissue extracts**

| **Acyl-carnitine species** | **Mean conc. (mM) saline ctrl** | **Std. dev. (mM) saline ctrl** | **Mean conc. (mM) DOX** | **Std. dev. (mM) DOX** | **p-value DOX vs saline (Student’s t-test)** |
| --- | --- | --- | --- | --- | --- |
| C0 | 0.590059 | 0.188245 | 0.921843 | 0.296287 | 0.004817 |
| C2 | 2.107055 | 0.556196 | 3.941691 | 1.656963 | 0.002117 |
| C3 | 0.428392 | 0.203835 | 0.422474 | 0.156547 | 0.939815 |
| C4 | 0.613081 | 0.254524 | 0.583042 | 0.13017 | 0.730784 |
| C6 | 0.007003 | 0.002652 | 0.010457 | 0.004384 | 0.035782 |
| C8 | 0.001963 | 0.001112 | 0.003385 | 0.001455 | 0.017227 |
| C10 | 0.001801 | 0.000868 | 0.003786 | 0.001687 | 0.002176 |
| C10:1 | 0.00081 | 0.000383 | 0.001444 | 0.000619 | 0.010887 |
| C12:1 | 0.001152 | 0.00066 | 0.003159 | 0.002494 | 0.017098 |
| C14:2 | 0.00091 | 0.000506 | 0.003623 | 0.002454 | 0.001629 |
| C12 | 0.001721 | 0.00071 | 0.003898 | 0.001851 | 0.001437 |
| C14:1 | 0.002872 | 0.001414 | 0.009244 | 0.005818 | 0.002827 |
| C14-OH | 0.000391 | 0.000377 | 0.000647 | 0.000285 | 0.085057 |
| C14 | 0.007922 | 0.00379 | 0.014649 | 0.006977 | 0.010198 |
| C15 | 0.000641 | 0.000353 | 0.000761 | 0.000237 | 0.360323 |
| C16:2 | 0.000802 | 0.00057 | 0.003389 | 0.002701 | 0.005137 |
| C16:1 | 0.006515 | 0.003987 | 0.011022 | 0.006199 | 0.054792 |
| C16:1-OH | 0.00024 | 0.000251 | 0.000371 | 0.000224 | 0.220885 |
| C16-OH | 0.00048 | 0.00059 | 0.000682 | 0.000326 | 0.331117 |
| C16 | 0.030742 | 0.014783 | 0.04105 | 0.011606 | 0.082549 |
| C17 | 0.000526 | 0.000188 | 0.000873 | 0.000412 | 0.018687 |
| C18:2 | 0.012923 | 0.01009 | 0.022232 | 0.009544 | 0.036816 |
| C18:1 | 0.031583 | 0.016394 | 0.056486 | 0.027477 | 0.017034 |
| C18-OH | 0.00026 | 0.000114 | 0.000634 | 0.000307 | 0.001013 |
| C18:2-OH | 0.000322 | 0.000487 | 0.000561 | 0.000329 | 0.192097 |
| C18:1-OH | 0.000682 | 0.000602 | 0.001252 | 0.000699 | 0.05246 |
| C18 | 0.008307 | 0.002468 | 0.01292 | 0.004944 | 0.011208 |
| C20:4 | 0.001862 | 0.001875 | 0.006198 | 0.004329 | 0.005891 |
| C20:2 | 0.001269 | 0.000642 | 0.002226 | 0.00095 | 0.011179 |
| C20:1 | 0.001925 | 0.000911 | 0.003712 | 0.001958 | 0.011814 |
| C:20 | 0.000673 | 0.000231 | 0.001106 | 0.000523 | 0.019682 |
